# Intravenous thrombolysis in patients with acute dizziness or imbalance and suspected ischemic stroke-systematic review

**DOI:** 10.1007/s00415-024-12782-7

**Published:** 2025-01-03

**Authors:** Jonathan A. Edlow, Alexander A. Tarnutzer

**Affiliations:** 1 Department of Emergency Medicine, Beth Israel Deaconess Medical Center, Boston, MA USA; 2 Harvard Medical School, Boston, MA USA; 3 Neurology, Cantonal Hospital of Baden, Baden, Switzerland; 4 Faculty of Medicine, University of Zurich, Zurich, Switzerland

**Keywords:** Vestibular, Acute ischemic stroke, Treatment, Intravenous thrombolysis, Systematic review, Recommendations

## Abstract

**Background:**

Correct identification of those patients presenting with an acute vestibular syndrome (AVS) or an acute imbalance syndrome (AIS) that have underlying posterior-circulation stroke (PCS) and thus may benefit from revascularization (intravenous thrombolysis (IVT), endovascular therapy (EVT)) is important. Treatment guidelines for AVS/AIS patients are lacking. We reviewed the evidence on acute treatment strategies in AVS/AIS focusing on predictors for IVT/EVT and outcome.

**Methods:**

We performed a systematic search (MEDLINE, Embase) to identify studies reporting on acute treatment in PCS presenting as AVS/AIS (PROSPERO-registration = CRD42024537272). Key parameters were extracted. Risk of bias was assessed (Downs-and-Black quality assessment checklist).

**Results:**

We identified 3883 citations and included seven study cohorts (*n* = 1000 patients including 950 ischemic strokes). Overall, 251/1000 patients (25.1%) received IVT; EVT was performed in 46/368 (12.5%). Acute vertigo/dizziness was reported in 295/1000 (29.5%) patients. AVS criteria were met in 186/407 (45.7%) patients evaluated, and AIS criteria in 82/346 (23.7%). IVT was reported in 71/195 (36.4%) AVS/AIS patients and EVT in 13/77 (16.9%) cases, whereas the door-to-needle time (DNT) was significantly longer for PCS compared to anterior-circulation stroke (90 ± 29min vs. 74 ± 30min, *p* = 0.003) in one study. DNT was similar in those patients presenting with AVS/AIS compared to all PCS presentations in another study (70 ± 39min (AVS/AIS) vs. 63 ± 35min (all)). An mRS $\le$ 2 after 90 days was noted in 68.4–69.6% of PCS. No outcome data were identified for AVS/AIS patients.

**Conclusions:**

Insufficient data exist to drive any firm recommendation about treating otherwise eligible patients with AVS/AIS with IVT/EVT and judgments must be made on a case-by-case basis. Further research on this specific patient group is needed.

**Supplementary Information:**

The online version contains supplementary material available at 10.1007/s00415-024-12782-7.

## Introduction

Between 3 and 4% of all US emergency departments (ED) visits are for a chief complaint of dizziness [1]. Half of those patients had general medical conditions, over 10% had neurological diagnoses and 4% (of the total) had cerebrovascular causes, mostly ischemic stroke [1]. Another study of 1666 adult ED patients with dizziness reported that stroke was found in 3.2% of patients, but only 0.7% of patients with isolated dizziness [2]. Patients with stroke causing isolated acute dizziness present with the acute vestibular syndrome (AVS – defined as acute-onset, continuous vertigo, dizziness or unsteadiness lasting days to weeks, generally accompanied by nausea/vomiting, gait imbalance, nystagmus, and motion intolerance) [3] or what has been termed the acute imbalance syndrome (AIS), in which the gait is more affected and nystagmus is usually absent.

Early identification of stroke in these patients allows for early initiation of secondary stroke prevention measures and vascular lesion-specific treatments to try to prevent a recurrent stroke or extension of the first one. A third potential reason is to use intravenous thrombolysis (IVT) or other vascular interventions in eligible patients [4]; however, data to support the use of IVT in this group of patients are sparse.

In the emergency department (ED), rapid identification of an acute stroke usually triggers a “Code Stroke” protocol whose main purpose is to rapidly acquire information to help decide which patients have strokes and are candidates for IVT. Data show that overall, IVT is safe and effective for posterior circulation stokes [5]. Randomized clinical trial data also make it clear that for patients with minor strokes, antiplatelet treatment is non-inferior to IVT in patients with NIHSS ≤ 5 [6, 7].

Current US and European clinical stroke guidelines recommend against IVT in patients with “non-disabling” minor stroke (NIHSS ≤ 5) who are otherwise eligible to be treated within 4.5 h [8, 9]. Prospective studies and meta-analyses have reported that IVT does not improve clinical outcomes in patients with minor strokes [10–13]. However, this group is heterogeneous and some studies have shown a signal that for patients with NIHSS of 3–5, IVT may improve functional outcomes [14, 15]. In another study of a prospectively collected registry of 35,113 stroke patients, 703 (2%) presented with a NINSS of 0–1 [16]. Compared to the 6316 patients with initial NIHSS of 2–5, the less-affected patients had more early neurological deterioration, more symptomatic intracranial hemorrhage (sICH), and lower rates of excellent outcomes (mRS 0–1). Other studies have reported that sICH seems to occur very infrequently in patients with small strokes although more often in patients with IVT compared to best medical management [10, 12, 13].

“Minor stroke” (defined by the NIHSS) and “non-disabling” stroke (defined functionally for that individual patient) are not equivalent. One definition of “disabling” includes “any deficit considered potentially disabling by the patient, family or the treating clinician” [17]. Thus, the same deficit (e.g., right arm clumsiness) might be non-disabling in a 75-year-old left-handed retiree compared to a 55-year-old right-handed surgeon.

Distinguishing patients with AVS due to stroke from those with a peripheral inner ear (e.g., acute unilateral vestibulopathy) or benign central (e.g., vestibular migraine) can be challenging. Making this distinction in the first 10 min of care, in order to decide whether or not to initiate a “Code Stroke” protocol is even more problematic. Additionally, after activating a “Code Stroke”, how should the decision be made to use IVT or not? A deficit that exists at minute 10 may not be present at day 90, even without IVT. To our knowledge, there are no prospective, randomized data on the effects of IVT on the outcomes of stroke patients.

The ideal data upon which to make an IVT treatment decision in patients with an AVS/AIS include the safety and efficacy of IVT in this specific setting, the natural history of these patients without treatment and the differences in safety and efficacy between IVT and anti-platelet agents. The absence of high-quality data results in practice variation and divergent opinion about which AVS/AIS patients should be treated with IVT [18].

Thus, the aim of this study was to systematically review the current evidence on acute treatment in central AVS/AIS related to ischemic stroke and to provide evidence-based recommendations.

## Methods

### Data sources and searches

We searched MEDLINE and Embase for articles, using the following strategies with the following components: (1) vertigo/dizziness, (2) acute treatment and outcome, and (3) central acute (transient) vestibular / imbalance syndrome (ischemic stroke). We also performed a manual search of reference lists from eligible articles and contacted corresponding authors where necessary. We did not seek to identify research abstracts from meeting proceedings or unpublished studies. We limited our search to articles published since 1995, when intravenous thrombolysis for ischemic stroke first became available. Our search was updated through April 15th, 2024. Being a systematic review of the literature, no ethical approval was necessary. This systematic review was prospectively registered at PROSPERO (CRD42024537272).

### Study selection and quality assessment

Articles were selected by two independent screeners using pre-determined inclusion criteria and a structured process (see Appendix 1 for details). Our focus was on studies examining acute treatment strategies in patients presenting to the ED with acute vertigo/dizziness or imbalance and suspected ischemic stroke within the treatment window for IVT and or EVT. We calculated interrater agreement on full-text inclusion using Cohen’s kappa [19]. We assessed the risk of bias for all studies using the Downs and Black quality assessment checklist and rated the overall quality of the study (excellent, good, fair, poor) [20]. As our systematic review did not include randomized controlled trials (RCTs), the checklist was modified and questions addressing criteria related to RCTs (questions 4, 5, 8, 13–15, 17, 19, 21–25, 27) were discarded. Thus, the scores for calculating overall quality of the study were adjusted accordingly.

### Data extraction, synthesis and analysis

We extracted epidemiologic data including patient age and gender, the study setting (prospective vs. retrospective, cross-sectional vs. interventional vs. observational), presenting symptoms and key benchmarks (NIHSS, symptom-onset to door time, door-to-needle time, modified Rankin Scale (mRS)) at presentation and fractions receiving intravenous thrombolysis (IVT) and/or endovascular treatment (EVT). Furthermore, we assessed the outcome and predictors for IVT / EVT. Due to the heterogeneity of the studies included, no meta-analysis across studies was possible. This systematic review is reported in accordance with PRISMA guidelines.

### Data availability

Source data used for the systematic review will be made available to others upon request to the corresponding author.

## Results

**Fig. 1:**
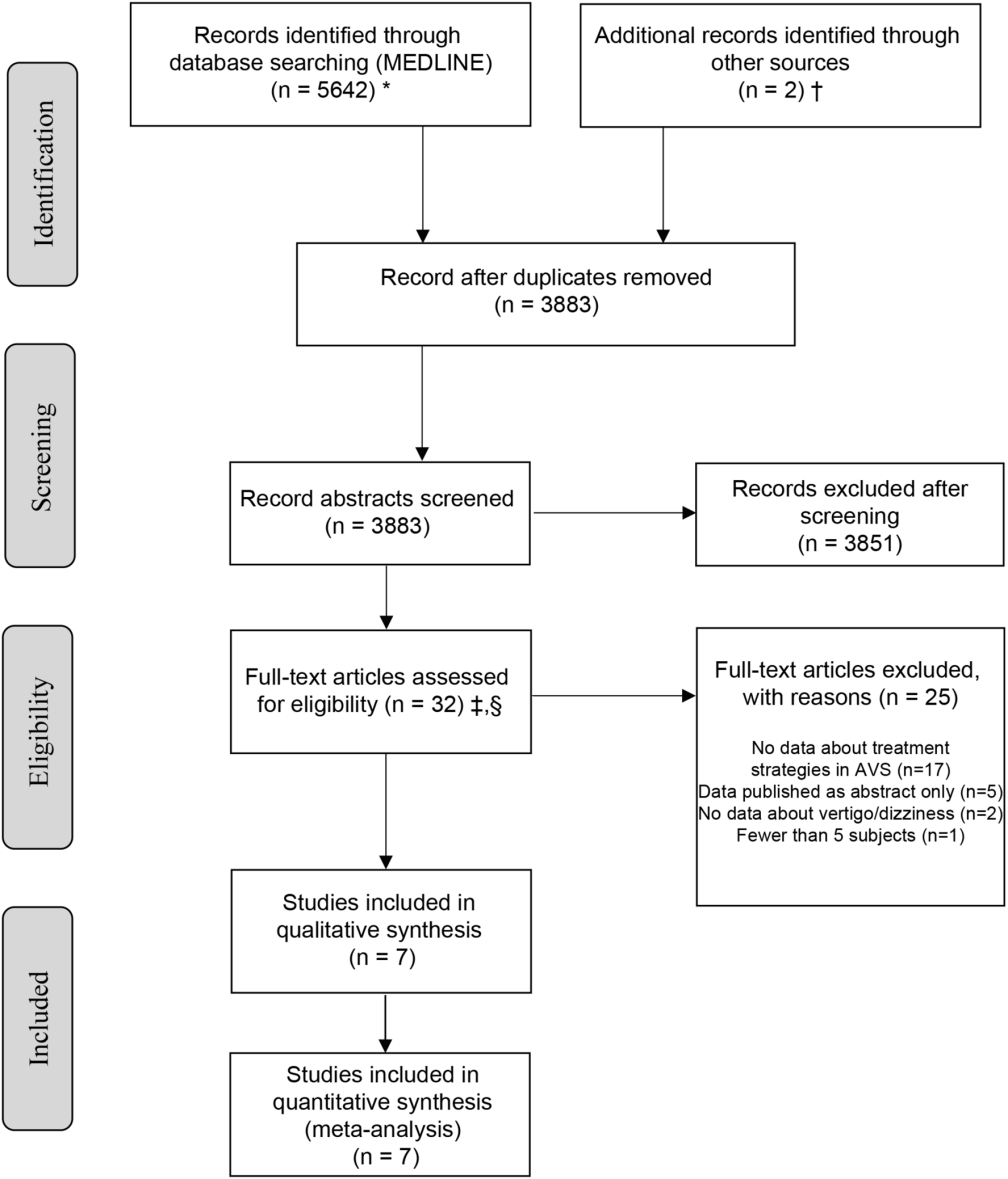
*MEDLINE was accessed via PubMed. † Individual hand search of citation lists from selected studies and investigator files identified 17 additional manuscripts for review. ‡ Abstracts coded as “yes” or “maybe” by at least one reviewer were included in full-text review. § After full-text evaluation by two reviewers, any differences were resolved by discussion and – if needed—adjudication by a third, independent reviewer

Our search identified 3883 unique citations, of which 3851 (99.2%) were excluded at the abstract level. Our independent raters had good initial agreement on inclusion of full-text manuscripts (kappa value 0.77). After resolving initial disagreements, 7/32 studies were considered eligible (Fig. 1—PRISMA flow chart), representing 0.2% of the total (Fig. 1).

The overall study quality using the Downs and Black quality assessment checklist was good in two studies, fair in four studies and poor in 1 study included.

### Characteristics of the study populations included

A total of 1000 patients were included from seven studies (range of sample size = 29 to 262 patients, overall, 40.3% females) that originated from 5 different countries. The study populations included varied substantially. Some focused on a diagnosis of acute ischemic stroke (a) without further restrictions [21, 22], b) with a presentation within 4.5h after symptom onset [23], or c) with IVT treatment [24]. Others focused on the presence of vestibular symptoms (acute vertigo, dizziness or imbalance) and a) referral to stroke service [25], or b) presentation within 4.5h after symptom onset [11, 18]. Acute ischemic stroke was by far the most frequent diagnosis (950/1000 cases), whereas other central causes, such as hemorrhagic stroke, TIA or transient vestibular episodes, were rare. Peripheral–vestibular causes were identified in 1.0% of patients, non-vestibular diagnoses were made in 1.1% of patients (see Table 1 for details).

**Table 1:** Key epidemiologic aspects

	N [studies, subjects]	%
** Gender**		
Women	[7, 403]	40.3
Men	[7, 597]	59.7
Total	[7, 1000]	100
** Diagnoses**		
** Central causes**		
Acute ischemic stroke [11, 18, 21, 23–25, 36]	[7, 950]	95.0
Acute hemorrhagic stroke [25]	[1, 6]	0.6
Other central causes* [18, 25]	[2, 23]	2.3
All central causes	[7, 979]	97.9
** Peripheral causes**		
Acute unilateral peripheral vestibulopathy [18, 25]	[2, 4]	0.4
Other peripheral causes† [25]	[1, 6]	0.6
All peripheral causes	[2, 10]	2.0
** Other, non-vestibular diagnoses‡** [25]	[1, 11]	1.1
** Study design**		
Prospective interventional [21, 24]	[2, 331]	33.1
Retrospective interventional [11, 18, 23, 36]	[4, 608]	60.8
Retrospective cross-sectional [25]	[1, 61]	6.1
** Study target group**		
** Patients with acute ischemic stroke**		
Anterior or posterior circulation	[1, 262]	26.2
Posterior circulation (cerebellum, pons)	[1, 79]	7.9
Posterior circulation (not further specified) presenting to the ED within 4.5h after symptom-onset [23]	[1, 228]	22.8
Anterior or posterior circulation that received IVT [24]	[1, 252]	25.2
** Patients with acute vertigo, dizziness or imbalance that**		
Presented to the ED and were referred to stroke service [25]	[1, 61]	6.1
Received a diagnosis of mild acute ischemic stroke and presented within 4.5h after symptom onset [11]	[1, 89]	8.9
Received a diagnosis of AVS/AIS and presented within 4.5h after symptom-onset [18]	[1, 29]	2.9
** Scores obtained**		
NIHSS at admission [11, 18, 21, 23–25, 36]	[7, 1000]	100
NIHSS at discharge [18]	[1, 29]	2.9
mRS at discharge [11, 24]	[2, 341]	34.1
mRS after 90 days [21, 23]	[2, 367]	36.7
** Timing reported**		
Symptom-to-door time [11, 18, 21, 23, 24]	[5, 677]	67.7
Door-to-needle time [21, 23, 24]	[3, 559]	55.9

^*^ Other central–vestibular diagnoses were transient ischemic attack (TIA) (*n* = 13), vestibular migraine (*n* = 4), Wernicke encephalopathy (*n* = 1), transient vestibular episode (TIA?) (*n* = 5)

^†^ Other peripheral–vestibular diagnoses were benign paroxysmal positional vertigo (*n* = 5). Diagnosis was not reported in 1 case

^‡^ Non-vestibular diagnoses were anxiety (*n* = 1), functional dizziness (*n* = 1), pre-syncope (*n* = 1). No specific diagnosis was made in 8 cases

*AIS* acute imbalance syndrome; *AVS* acute vestibular syndrome; *ED* emergency department; *mRS* modified Rankin scale; *NIHSS* National Institutes of Health Stroke Scale

The study setting was the ED in all studies and the study design was retrospective in 5 studies and prospective in 2 studies. Both prospective studies were interventional, i.e., assessed the impact of IVT and/or EVT. Among the retrospective studies, four were interventional and one study was observational cross-sectional (for details see Table 1).

### Presenting symptoms and scores

Clinical presentation at admission strongly depended on the patient population studied, whereas some studies included patients based on MR-confirmed stroke, others required certain clinical symptoms and/or findings (see Table 2). Acute vertigo or dizziness was reported in 295/1000 (29.5%) patients, and acute severe imbalance (i.e., inability to stand unassisted) was noted in 59/118 (50%) patients. Only a subset of patients was evaluated for a possible diagnosis of AVS (407/1000, 40.7%) or AIS (346/1000, 34.6%). Diagnostic criteria for an AVS were met in 186/407 (45.7%) patients and 82/346 (23.7%) patients were classified as AIS.

**Table 2:** Clinical findings and treatment response

	Patients with finding / all patients assessed (*n*)	%
** Clinical presentation**		
Acute vertigo or dizziness [11, 18, 21, 23–25, 36]	295/1000	29.5
Severe (grade 3) truncal instability [11, 18]	59/118	50.0
Acute vestibular syndrome (AVS) [11, 18, 23, 25]	186/407	45.7
Acute imbalance syndrome (AIS) [11, 18, 23]	82/346	23.7
** NIHSS at admission**	**Value**	
** Acute ischemic stroke**		
Stroke team activated [36]	7.7 ± 6.9 (mean ± 1SD)	
Stroke team not activated [36]	3.6 ± 5.7 (mean ± 1SD)	
Cerebellar or pontine stroke [21]	4 (median, no IQR reported)	
PCS, presenting within 4.5h after symptom onset [23]	3 [1–13] and 5 [2–10] (median [inter-quartile range]) *	
** Acute vestibular syndrome / acute imbalance syndrome**		
AVS / AIS due to PCS presenting within 4.5h after symptom onset [23]	2 [1–6] and 2 [1–5] (median [inter-quartile range]) *	
AVS / AIS due to ischemic stroke receiving IVT [18]	1.9 ± 1.2 (mean ± 1SD)	
AVS / AIS due to ischemic stroke receiving no IVT [18]	0.8 ± 1.0 (mean ± 1SD)	
** Door-to-needle time (DNT)**	**Time (mean ± 1SD)**	
Acute ischemic stroke, not further specified [23]	63 ± 35min	
PCS presenting as AVS / AIS [23]	70 ± 39min	
PCS (no further specified) [24]	90 ± 29min	
Acute cerebellar stroke [21]	21min (no SD reported)	
Acute brainstem (pontine) stroke [21]	35min (no SD reported)	
** Acute reperfusion treatments performed**	** Patients with finding /** **all patients assessed **(*n*)	**%**
Intravenous thrombolysis (IVT) [11, 18, 21, 23–25, 36]	251/1000	25.1
IVT in AVS/AIS [11, 18, 23]	71/195	36.4
Endovascular treatment (EVT) [21, 23, 25]	46/368	12.5
EVT in AVS/AIS [23]	13/77	16.9
** Favorable outcome (i.e., mRS** $\le$ **2 at 90 days)**	** Patients with finding / all patients assessed** (n)	%
Acute cerebellar stroke [21]	23/43	53.5
Acute brainstem (pontine) stroke [21]	22/36	61.1
PCS presenting as AVS / AIS [23]	60/77	77.9

^*^ NIHSS median values were reported separately for two observation periods with distinct code stroke criteria

*AIS* acute imbalance syndrome; *AVS* acute vestibular syndrome; *EVT* endovascular treatment; *IQR* inter-quartile range; *IVT* intravenous thrombolysis; *mRS* modified Rankin Scale; *PCS* posterior circulation stroke; *SD* standard deviation

The NIHSS at admission (reported in all seven studies) in posterior circulation stroke (PCS) patients varied substantially depending on the patient population (see Table 2). The NIHSS was significantly higher in patients with anterior circulation strokes (ACS—median 13, interquartile range 8–19) compared to those with posterior circulation strokes (PCS—median 6, interquartile range 4–16) in one study [24]. Among patients with PCS, the NIHSS was lower in those patients presenting as AVS or AIS compared to all PCS presentations (median of 2 vs. 3–5 median) in another study [23]. Furthermore, the NIHSS was significantly lower in those patients presenting with AVS or AIS that received no IVT compared to those that received IVT (0.8 ± 1.0 vs. 1.9 ± 1.2, *p* = 0.004, mean ± 1 standard deviation) in one study [18].

### Treatment strategies

Overall, 25.1% (251/1000) of all patients included in this systematic review received IVT. Numbers on EVT were available from three studies [21, 23, 25], with treatment performed in 12.5% (46/368) of patients. Focusing on those patients presenting with AVS or AIS within the treatment window (i.e., within 4.5 h after symptom onset), IVT was reported in 71/195 (36.4%) cases from three studies [11, 18, 23] and EVT in 13/77 (16.9%) cases from a single study [23]. Door-to-needle time was reported in three studies only [21, 23, 24], whereas the door-to-needle time (DNT) was significantly longer for PCS compared to ACS (90 ± 29min vs. 74 ± 30min, *p* = 0.003) in one study. [24] DNT was similar in those patients presenting with AVS or AIS compared to all PCS presentations in another study (70 ± 39min (AVS/AIS) vs. 63 ± 35min (all) [23].

### Outcome

The mRS after 90 days (considered the gold standard for assessing outcome after stroke) was reported in two studies [21, 23]. In other three studies, only the NIHSS [18] or the mRS [11, 24] at discharge was provided. A favorable mRS after 90 days (defined as an mRS $\le$ 2) was noted in 68.4–69.6% of all PCS. [21, 23]

## Discussion

The ideal data that would help inform rational decision-making around giving or not giving IVT to patients with acute ischemic stroke that present as an AVS or an AIS would be from a randomized double-blinded trial. Short of that, examining outcomes of a homogenous population of those patients from large stroke registries would be useful. Neither class of data is currently available.

We have tried to analyze what data do exist in an attempt to inform this decision, but several variables impact their interpretation. These include the speed and accuracy of early identification of stroke patients presenting as isolated dizziness in the ED, when to activate a “Code Stroke”, the intermediate to long-term natural history of these patients untreated with IVT, and the efficacy and complications when they are treated with IVT versus anti-platelet agents.

Identification of acutely dizzy patients with a stroke in the ED is important. Although it is clear that emergency clinicians can learn to effectively use elements of the HINTS exam in this setting, [26–29] it is equally clear that current routine use in the ED is suboptimal. [30] Because these patients usually have lower NIHSSs or even zero on the NIHSS [31] and presence of non-localizing symptoms, such as headache, nausea and vomiting, dizziness, truncal ataxia and nystagmus, [22, 24, 31] early recognition can be difficult. One study reported that of 61 patients presenting with vestibular symptoms who were referred for a suspected stroke, over half had ischemic strokes, but none were treated with IVT [25].

In one study of 29 patients with an AVS or AIS presentation who were suspected of having a stroke and admitted to a stroke unit, 15 were treated with IVT and 14 were not. Treated patients were more likely to have “disabling” vestibular symptoms defined as the “perception” of severe dizziness or vertigo and inability to stand unsupported. [18] They were also more likely to have “focal symptoms” such as dysarthria or hemiataxia, but patients with overt symptoms, such as hemiplegia, hemianopia, aphasia and anarthria, were excluded from the study. The mean NIHSS for treated patients was 1.9 (range 0–4) and in the untreated patients 0.8 (range 0–3). Of the 15 IVT-treated patients, at discharge, 13 had a stroke diagnosis and 1 each, vestibular neuritis and transient ischemic attack (TIA). Of the 14 non-IVT-treated patients, 8 had a stroke and 1 and 4, respectively, had neuritis and TIA. [18] No patient in either group had a hemorrhagic complication and their clinical outcomes were not significantly different. Although the numbers were low, the authors concluded that IVT for patients presenting with AVS/AIS neither helped nor harmed.

In a second retrospective study of 89 consecutive patients with mild ischemic strokes (NIHSS < 5) with primary vestibular symptoms, and who presented to an ED within 4.5 h of symptom onset, 47 were treated with IVT and 42 were not [11]. The two groups were demographically equal. Treated patients had shorter symptom onset-to-door times, higher NIHSSs and more often “disabling” deficits including dysarthria, facial weakness, unilateral tongue weakness, limb weakness or sensory loss. Treated patients had more multi-modal imaging done (CTA and CTP) prior to deciding on IVT. Treated patients were more likely to have limb weakness or hemiplegia. There were no statistically significant differences in the mRS between the IVT treated and untreated patients on discharge from the hospital (1 with a range of 0–2 for both groups). Brain hemorrhage occurred in 4/47 (8.5%) of the treated patients and 2/42 (4.8%) of untreated patients (*p* = 0.680). [11]

Another study that investigated the effect of changing Code Stroke activation from a more-inclusive one to a less-inclusive one (dropping acute dizziness or vertigo as a criterion for activation) also reported that the change did not negatively impact patient clinical outcomes as measured by the mRS at 3 months. [23] Nearly 96% of the AVS/AIS patients had a mRS of 0–1. Despite the fact that a Code Stroke was activated in nearly 25% of patients and that large vessel disease was found in over one third of patients, IVT was only performed in 1 (of the total 45). One important finding in this study was that of the 23 patients who had prodromal vestibular symptoms and then returned with worse deficits, 9 (39%) occurred within 3 h and nearly 80% returned within 24 h.[23] This underscores the importance of recognizing and managing posterior circulation TIAs that present with isolated vestibular symptoms [32, 33]. One other finding was that 28/42 (67%) of the untreated patients were misdiagnosed in the ED as non-stroke.

Because the NIHSSs of patients with PCS presenting as an AVS/AIS are low, [24] some investigators have recommended that the “decision-making process for [IVT] in PCS should focus on individual symptoms rather than” the NIHSS or DWI lesion volume, which did not correlate with the NIHSS in their patients with PCS. [21] Others have proposed a modified NIHSS that includes an assessment of gait/truncal ataxia and bulbar signs (including dysphagia, diplopia, nystagmus and Horner syndrome) [34]. These findings are helpful to identify posterior circulation stroke patients presenting with low NIHSS who may be at higher risk of poor outcome and might benefit from reperfusion therapies.

Regarding anti-platelet treatment for patients with minor strokes, the PRISMS trial compared IVT with aspirin in patients whose deficits were “not clearly disabling” who were treated ≤ 3 h from symptom onset [7]. The ARAMIS study compared IVT with clopidogrel in patients treated within 4.5 h [6]. Large registry data also show lack of superiority for IVT in this group [35]. None of these studies reported data on the subset of patients who presented with acute dizziness, vertigo or imbalance.

### Limitations

The primary limitation is the absence of high-quality data in the literature. We were unable to perform a meta-analysis due to the heterogenous inclusion criteria among the studies that we did identify and include. There was further heterogeneity in published results in that some studies included all acute stroke patients within the IVT window and others only reported on those with AVS/AIS patients.

## Conclusions

Currently, insufficient data exist to drive any firm recommendation about treating otherwise eligible patients with the acute vestibular syndrome/acute imbalance syndrome with IV thrombolysis and judgments must be made on a case-by-case basis. Further research on this specific patient group is needed.

## Supplementary Information

Below is the link to the electronic supplementary material.

Supplementary file1 (DOCX 22 KB)

## Data Availability

Source data used for the systematic review will be made available to others upon request to the corresponding author.
