# Supplementary material for: Intravenous thrombolysis in patients with acute dizziness or imbalance and suspected ischemic stroke-systematic review
Source: J Neurol. 2025 Jan 3;272(1):91. doi: 10.1007/s00415-024-12782-7 (PMC11698810; doi:10.1007/s00415-024-12782-7)
Supplement: Supplementary file 1 — Supplementary file1 (DOCX 22 KB) [file 415_2024_12782_MOESM1_ESM.docx]

**Intravenous thrombolysis in patients with acute dizziness or imbalance and suspected ischemic stroke-systematic review**

**Journal of Neurology**

Jonathan A. Edlow (1, 2) and Alexander A. Tarnutzer (3, 4)

1. Department of Emergency Medicine, Beth Israel Deaconess Medical Center, Boston, MA, USA (jedlow@bidmc.harvard.edu)
2. Harvard Medical School, Boston, MA, USA
3. Neurology, Cantonal Hospital of Baden, Baden, Switzerland ([alexander.tarnutzer@access.uzh.ch](mailto:alexander.tarnutzer@access.uzh.ch))
4. Faculty of Medicine, University of Zurich, Zurich, Switzerland

**Short title:** IV thrombolysis in acute vestibular syndrome

**Corresponding author:**

Prof. Alexander A. Tarnutzer

Neurology, Cantonal Hospital of Baden

5404 Baden, Switzerland

Email: [alexander.tarnutzer@access.uzh.ch](mailto:alexander.tarnutzer@access.uzh.ch)

phone: +41 56 486 16 10

ORCID: [0000-0002-6984-6958](https://orcid.org/0000-0002-6984-6958)

## **Supplementary file 1- electronic search strategy, coding-scheme for the systematic review and data analysis**

**The search strategy was designed by a investigator with relevant domain expertise in neurology (AAT) and an expert in emergency medicine (JAE).**

**We searched MEDLINE and Embase for articles, using the following strategies with the following components: (1) vertigo/dizziness, (2) acute treatment and outcome, and (3) central acute (transient) vestibular syndrome (ischemic stroke). We also performed a manual search of reference lists from eligible articles and contacted corresponding authors where necessary. We did not seek to identify research abstracts from meeting proceedings or unpublished studies.**

AGGREGATED/COMPOSITE VERSION (April 15^th^ 2024) [PubMed ~1944 abstracts; EMBASE ~3698]

**Pubmed**

((dizz*[tiab] OR vertigo[tiab] OR vestibular[tiab] OR imbalance[tiab]) AND (management[tiab] OR treatment*[tiab] OR thrombolysis*[tiab] OR endovascular*[tiab] OR thrombectomy*[tiab] OR aspirin*[tiab] OR clopidogrel[tiab] OR code stroke[tiab] OR outcome*[tiab] OR guideline*[tiab] OR recommendation*[tiab] OR prognosis[tiab] OR decision*[tiab] OR consensus[tiab]) AND (cerebrovascular[tiab] OR stroke*[tiab] OR vertebrobasilar insufficiency[tiab] OR TIA[tiab] OR transient ischemic attack[tiab] OR transient neurologic* deficit[tiab]) AND 1995/01/01:2024/04/15[dp])

DISAGGREGATED VERSION [to demonstrate overall structure] (April 15, 2024)

((dizz*[tiab] OR vertigo[tiab] OR vestibular[tiab] OR imbalance[tiab])

AND

(treatment*[tiab] OR thrombolysis*[tiab] OR endovascular*[tiab] OR thrombectomy*[tiab] OR aspirin*[tiab] OR clopidogrel[tiab] OR code stroke[tiab] OR outcome*[tiab] OR guideline*[tiab] OR recommendation*[tiab] OR prognosis[tiab] OR decision*[tiab] OR consensus[tiab])

AND

(cerebrovascular[tiab] OR stroke*[tiab] OR cerebellar[tiab] OR hemorrhag*[tiab] OR haemorrhag*[tiab] OR vertebrobasilar insufficiency[tiab] OR TIA[tiab] OR transient ischemic attack[tiab] OR transient neurologic* deficit[tiab])

AND 1995/01/01:2024/04/15[dp])

**Embase:**

((dizz*:ab,ti OR vertigo:ab,ti OR vestibular:ab,ti OR imbalance:ab,ti) AND (management:ab,ti OR treatment*:ab,ti OR thrombolysis*:ab,ti OR endovascular*:ab,ti OR thrombectomy*:ab,ti OR aspirin*:ab,ti OR clopidogrel:ab,ti OR ‘code stroke’:ab,ti OR outcome*:ab,ti OR guideline*:ab,ti OR recommendation*:ab,ti OR prognosis:ab,ti OR decision*:ab,ti OR consensus:ab,ti) AND (cerebrovascular:ab,ti OR stroke*:ab,ti OR ‘vertebrobasilar insufficiency’:ab,ti OR TIA:ab,ti OR ‘transient ischemic attack’:ab,ti OR ‘transient neurologic* deficit’:ab,ti) AND [01-01-1995]/sd)

### Inclusion and exclusion rules for abstracts & full-text manuscripts

**All gathered literature was subject to title/abstract screening by two independent reviewers (AAT/JAE). Abstract review coding rules are provided below. Full-text screening was applied to all citations considered eligible or possibly eligible by at least one reviewer. Two independent reviewers (AAT/JAE) determined whether full-text manuscripts are eligible and, if not, provided a reason for exclusion (see full-text review coding rules below). Differences were resolved by discussion and consensus. JAE and AAT completed a hand search of the reference lists of selected articles for additional citations. For citations identified by hand search, the full process was repeated iteratively until no additional manuscripts were found for inclusion. Inter-rater agreement on full-text inclusion was calculated using Cohen’s kappa.^1^**

Abstract Reasons for Exclusion

| 0 | not humans | No reasonable prospect that the study reports on human subjects |
| --- | --- | --- |
| 1 | not dizziness | no reasonable prospect that the study includes data about dizziness or vertigo |
| 2 | not acute | no reasonable prospect that the study includes data about *acute* (<72 hours) dizziness or vertigo |
| 3 | not treatment | no reasonable prospect that the study includes data about treatment strategies or outcome of acute central (specifically stroke, TIA) disorders |
| 4 | <5 cases | fewer than 5 subjects (total participants reported, including cases and controls) |
| 5 | abstract only | only abstract available (from poster presentation or talk at conference) |
| 6 | other | any other reason abstract is not included |

Full-Text Reasons for Exclusion

| 0 | not humans | No reasonable prospect that the study reports on human subjects |
| --- | --- | --- |
| 1 | not dizziness | no reasonable prospect that the study includes data about dizziness or vertigo |
| 2 | not acute | no reasonable prospect that the study includes data about *acute* (<72 hours) dizziness or vertigo |
| 3 | not treatment | no reasonable prospect that the study includes data about treatment strategies or outcome of acute central (specifically stroke, TIA) disorders |
| 4 | <5 cases | fewer than 5 subjects (total participants reported, including cases and controls) |
| 5 | abstract only | only abstract available (from poster presentation or talk at conference) |

**References**

1. Cohen J. A coefficient for agreement for nominal scales. *Educ Psychol Meas.* 1960;20:37-46.
